# Weathering the storm: youth vulnerability and resilience during the climate crisis

**DOI:** 10.1038/s44168-025-00288-5

**Published:** 2025-09-06

**Authors:** John A. Pollock, Brinley Kantorski

**Affiliations:** 1 The Partnership in Education, Duquesne University; Pittsburgh, Pittsburgh, PA USA; 2 Department of Biological Science, School of Science & Engineering, Duquesne University; Pittsburgh, Pittsburgh, PA USA

**Keywords:** Climate sciences, Environmental sciences, Environmental social sciences, Scientific community, Social sciences

## Abstract

Teens are experiencing an increase in the incidence of anxiety and depression. Climate change adds uncertainty. Dire predictions and unknown impacts contribute to teens' worldview, increasing concerns that add to their normal stressors and anxiety; and for some, this becomes overwhelming. Here, we present a new perspective on teen mental health education and the impact of learning about climate change. We conclude that a comprehensive education can integrate the facts of global climate change, along with the progress in climate mitigation together with mental health education.

## Introduction

The global climate is changing because of humanity’s use of fossil fuels. Yet overall, only 45% of Americans understand that human activity is a significant contributor to climate change, with this perspective greatly influenced by political leanings and age demographic^1^. Nonetheless, climate change remains the single greatest threat to humans’ physical and mental health^2,3^. The significance of this threat arises from the insidious penetration of climate change into all aspects of life through changes in air temperature, air quality, food and water availability and infectious disease among other challenges^4^. While this is happening, it is evident that rising temperatures are having direct effects on the mental and physical health of children^5^. In fact, a recent study explored the relationship between catastrophic climate events and emergency department visits for depression and anxiety in Houston, Texas finding a surge in visits associated with depression and anxiety, especially for children under 18 years of age^6^. In another study, Vercammen and colleagues^7^ assessed over 2800 US youth regarding their sense of their own mental health as well as psychological distress and their sense of agency over climate change. The approach is based on the Changing Worlds Survey^8,9^. Their overall observation is that youth are already struggling with the obvious reality of climate change and that it is directly impacting their sense of the future. Additional studies also identify the gap between mental health interventions and climate policy^10^ and others focus on building overall resilience to climate change, recognizing that it requires an added focus on mental health and well-being^11^. In fact, a survey of Americans found that nearly 90% of those aged 18-29 already expect to make sacrifices in their lives due to climate change^1^. This perspective feeds into the underlying concerns related to the changing climate and its impact on the environment. These concerns, are now being called ‘eco-anxiety’, demonstrating the direct links between mental health and climate change^2^.

## Discussion

Throughout human history, the adolescent transition from childhood to adulthood has been recognized as a time of dramatic physical, behavioral and emotional change, which often results in elevated levels of stress and anxiety^12^. This is recognized as a result of changes in brain growth, myelination and hormonal changes that impact emotions and reactivity^13^. Although most teens learn to cope and develop resilience, adolescence is a time when clinical psychiatric disorders may arise^12^. The youth of today were born into a rapidly warming climate with news broadcasts and sensationalized social media commentary about obvious disasters (extreme heatwaves, wildfires, super-storms and floods) counterposed with two-sided political commentary which leaves them wondering what to believe. As the media storm progresses, they often are left with little to no positive and hopeful messaging about acceptance of the realities that we are facing and mitigation plans that can provide a meaningful response. With adolescents already at increased risk of mental health issues, youth now face the extraordinary challenges caused by a changing climate that disproportionately exacerbate the threat to their mental health^14^ This new reality is something that many previous generations never had to think about^15^. In the past, stressful events such as famine and pandemics, civil and world wars, and even the Great Depression all had a tangible end point that could be imagined. Climate change differs from these challenges of the past, in that it is difficult for people to envision a positive endpoint to climate change. Let us not forget, that war, famine and social strife persist across the globe today and are not simply relegated to history books. Yet, added to these persistent challenges, it is now clear that without management, and considerable mitigation efforts, global climate change will have increasingly negative impacts on all of humanity.

The cognitive and emotional issues associated with climate change natural disasters are unique and present novel stressors that elevate existing anxieties. Teenagers have fewer life experiences than adults, which affects their perspective and context for considering climate change impacts. In some cases, they may lack the perspective that comes from realizing potential of recovery and rebuilding. Without that perspective, for some teens, the perceived total loss caused by a climate challenge can result in despair^16^. It is important to address this despair and its corresponding stress directly, because the consequences of unmitigated stressful ideation can be poor performance in almost all aspects of adolescent life: school, extracurriculars, self-image, and personal relationships. Lacking a comprehensive picture and contextual knowledge can further fuel negative feelings that can then spiral out of control, leading to serious mental health issues that can include depression, self-harm, drug abuse, or suicidal ideation and even death^17^.

Studies by the Center for Disease Control and Prevention^18^ and the Pew Research Center^19^ and others reveal that more than a third of high school students already self-report experiencing mental health challenges, a significant increase from a decade ago. Taken together, a growing body of literature points to direct impacts of climate change on the mental health of children and adolescents as well as the need for robust education on both issues^20,21^. However, asking students to study only the science and consequences of the changing climate and its impacts can, in and of itself, be very depressing. Instead, allowing students to learn the science of climate change so that it is relatable to their own lives allows them to contextualize this new knowledge. By teaching climate science in this way, integrating lessons on mindfulness and the physiological basis of stress and stress management will help students to build a new tool kit of life skills that will continue to inform and support them^22,23^. For example, a college curriculum has recently been deployed that aims to build psychosocial climate resilience^24^. With 190 students engaged in a 10-week course for credit, a significant improvement in mental health and confidence for self-efficacy for climate action was achieved. This sort of demonstration reveals that programmatic interventions can have a positive impact and that course materials that focus on basic skills for supporting mental health combined with experiential learning that empowers students to see that climate solutions are achievable, are collectively effective. In another approach, Benoit and co-workers^14^ have tested using brief ‘selfie’ videos to convey messages about climate change, finding that positive messaging could increase teen ‘hopefulness and a sense of agency’. This intervention, however, did not decrease climate anxiety. Nonetheless, this shows that intentional message can have a useful impact. The challenge is to adapt these and other approaches for use in middle and high school. An international team of editors and contributors is addressing this challenge head on. In a new monograph, they strive to both reveal the degree of anxiety and why the anxiety exists, as well as strategies for building a comprehensive learning environment^25^. Overall, we see that a starting point can be the recognition that mental health is of equal importance to physical health. In schools across the US, physical health isn’t taught in one learning block. It is weekly physical education time, health classes, units on diet and nutrition, and even information provided in cafeteria breakfast and lunch programs. All of these are done in age-appropriate ways across all the grades from kindergarten to 12^th^ grade. Mental health needs to be addressed in a similar manner.

Helping students to learn self-management of “everyday” stressors and challenges based on their own experiences and worldview will help them to establish empowered coping skills. Using these skills to engage with the practice of intentional mindfulness, good sleep habits, attention to physical activity, and diet all help to build mental resiliency. These cultivated skills can help students deal with mental health challenges that would be considered developmentally typical or pre-clinical, but that are nonetheless of significant and real importance to them in that moment. These challenges and concerns need to be respected by adults, teachers and parents. Failure to appropriately cope with challenges in childhood and adolescence can result in persistent trauma responses and chronic anxiety in adulthood^26^. An additional concern is that some adolescents will do all that they can to hide their mental health pain to avoid embarrassment or stigma. Consider an example of a teen with an earache. You cannot tell that they have an earache until they tell you, but then a healthcare professional can be engaged to provide an appropriate treatment. There is no stigma associated with seeking help when one is physically ill, or in pain, why then should one feel differently about mental illness? When a teen has a mental health challenge, again no one can tell by looking. But if there is a perceived stigma on seeking help, the student may go on suffering needlessly. As such, society has a responsibility to destigmatize mental health and to normalize seeking help from professionals when unwell. This is imperative across all ages, not just among youth. Additionally, teens can find empowerment in realizing that they can strengthen their mental resilience just like strengthening their physical health. They can also learn to recognize when self-care isn’t enough, and when they (or their friends) need the help of a mental health professional.

Some initiatives are bringing climate change and health learning together in undergraduate and graduate medical programs^24,27^, yet more needs to be crafted for grade school, middle and high school student curriculum. Certain youth programs may be aligned with the solutions outlined by McGorry and colleagues, which emphasizes a spectrum of interventions^28,29^. These include programs that strive to establish greater teacher confidence and knowledge about climate through intentional professional development. The goal is to shift classroom practice and to enhance student empowerment that ultimately leads to systemic change in how climate change is taught^30^. These researchers found they could improve teacher confidence in their instructions about climate change, and the teachers increased use of action-oriented instruction. While these defining examples of intentional curriculum development are helping to show the way, there is a moral imperative to do more that will link the pedagogy of these two critically important topics to accessible lessons that teachers can teach.

The need for an intentional plan for raising awareness of mental health education for self-awareness and self-care is greatest for adolescents in communities where there is already a higher incidence of anxiety or depression, which correlates with income inequality and other social barriers to personal success. If a community is already struggling, then a climate related challenge can intensify the incidence of anxiety and depression. It is these communities that are typically least prepared to respond to climate related impacts and are least equipped to mitigate those pressures. Government can sometimes help, but more often now non-governmental organizations need to be engaged, stepping in to help identify specific needs and then mobilize action. This can be facilitated by groups like the ICLEI – Local Governments for Sustainability (https://iclei.org/)^31^ and Rockefeller Foundation 100 Resilient Cities initiative (https://www.rockefellerfoundation.org/100-resilient-cities/)^32^ which are among the growing number of grass-root organizations that are taking action.

A starting point for action in developing new curriculum that represents a more comprehensive learning opportunity about climate change paired with learning about mental health will be built on reliable resources and facts. Some of the resources for learning about the scientific facts of climate change may continue to be available from the National Oceanic & Atmospheric Administration (NOAA) Global Climate Dashboard (https://www.climate.gov/climatedashboard). Additional sources of accurate, un-biased data and information are also available from a variety of sources^33^, including among others the Climate Change Knowledge Portal facilitated by the World Bank (https://climateknowledgeportal.worldbank.org/)^34^, the European Space Agency (ESA) Climate Change Initiative (CCI) (https://climate.esa.int/en/)^35^. The Our World in Data (https://ourworldindata.org/) project also hosts climate data^36^. Other resources include the Center for Disease Control and Prevention (https://www.cdc.gov/climate-health), which has historically hosted web pages that provide information on the impacts of climate change on various areas of human health. Other reliable non-governmental sources also need to be considered including among others, the World Health Organization Climate Change & Health program (https://www.who.int/teams/environment-climate-change-and-health), Our World in Data (https://ourworldindata.org/), The Lancet Countdown: Tracking Progress on Health and Climate Change (https://lancetcountdown.org/)^37^, and the National Academy of Medicine (USA) Climate Network (https://nam.edu/our-work/programs/climate-and-health/)^38^. This highlights the need to intentionally diversify the sourcing of reliable information and current guidance on issues climate and health.

It is imperative that thoughtful integration of positive progress in mitigating climate change across several domains be emphasized^39^. While students delve into the interlinked facets of climate science and its resulting impacts on society, they can simultaneously learn about some of the positive strides that have been made in relation to climate and environmental issues. Even when environmental damage^7^ is profound, awareness, social engagement and public pressure for change can have a positive effect^40,41^. Examples include the impact of Rachel Carson’s book, *Silent Spring*, which helped bring about the 1972 ban on DDT, or the nearly forgotten issue of ‘acid rain’ in the 1970s and 80s^40^. Caused by harmful emissions from burning coal, public pressure around the world resulted in new standards, which led to drastic reductions in acid rain and its negative impacts. Public awareness and governmental action can bring about significant and positive progress in solving problems.

The current wealth of data on climate change, its impacts and projections, make it clear that at the same time that we face important global climate challenges that were brought on by human habits, the direst predictions are not inevitable. Clearly much more needs to be done, but a positive way forward can be visualized.

Weathering the storm of climate change demands emotional preparedness and resilience, especially among youth. As adolescents confront an uncertain future, the integration of climate science and mental health education becomes not only a necessity, but a moral imperative. By empowering students with knowledge about the physiology of stress, homeostasis and allostasis^42^ while destigmatizing mental health struggles, and highlighting examples of environmental progress, we can nurture a generation that is not only informed but also resilient, hopeful, and ready to lead. The time to invest in comprehensive, youth-centered educational and policy frameworks is now, because safeguarding adolescent mental health is fundamental to building a sustainable future for all.

## Limitations

A limitation is that our observations presented here are not based on original data, but rather a synthesis of existing perspectives. Additional studies are needed to both more deeply understand the challenges of effective teaching that integrates the learning goals of mental health resiliency and an understanding of climate change.

## Conclusion

Modernizing curriculum, pedagogy and classroom practice are usually slow and sometime tortuous in the US educational system with its more than 13,000 distinct locally managed school districts. However, the facts are evident that mental health issues and climate change are a real and present challenge in every community and cannot be ignored. Whether or not curriculum standards are set nationally or by states or not at all, there is a moral imperative to help students understand their world and themselves amid the challenges of a changing climate. Acknowledgement of the issues and need is the first step that many teachers are already striving to accommodate on their own, often only with their good sense and their ability to adopt found resources. A call for action is warranted so that a comprehensive assessment of the range of needs for each community is acknowledged, appropriately resourced and supported with comprehensive programming and teacher professional development.

## Data Availability

No datasets were generated or analysed during the current study.
